# Less is more in language production: an information-theoretic analysis of agrammatism in primary progressive aphasia

**DOI:** 10.1093/braincomms/fcad136

**Published:** 2023-04-25

**Authors:** Neguine Rezaii, Boyu Ren, Megan Quimby, Daisy Hochberg, Bradford C Dickerson

**Affiliations:** Frontotemporal Disorders Unit, Department of Neurology, Massachusetts General Hospital, Harvard Medical School, Boston, MA 02129, USA; Department of Psychiatry, Laboratory for Psychiatric Biostatistics, McLean Hospital, Belmont, MA 02478, USA; Frontotemporal Disorders Unit, Department of Neurology, Massachusetts General Hospital, Harvard Medical School, Boston, MA 02129, USA; Frontotemporal Disorders Unit, Department of Neurology, Massachusetts General Hospital, Harvard Medical School, Boston, MA 02129, USA; Frontotemporal Disorders Unit, Department of Neurology, Massachusetts General Hospital, Harvard Medical School, Boston, MA 02129, USA

**Keywords:** agrammatism, primary progressive aphasia, word frequency, entropy, information theory

## Abstract

Agrammatism is a disorder of language production characterized by short, simplified sentences, the omission of function words, an increased use of nouns over verbs and a higher use of heavy verbs. Despite observing these phenomena for decades, the accounts of agrammatism have not converged. Here, we propose and test the hypothesis that the lexical profile of agrammatism results from a process that opts for words with a lower frequency of occurrence to increase lexical information. Furthermore, we hypothesize that this process is a compensatory response to patients’ core deficit in producing long, complex sentences. In this cross-sectional study, we analysed speech samples of patients with primary progressive aphasia (*n* = 100) and healthy speakers (*n* = 65) as they described a picture. The patient cohort included 34 individuals with the non-fluent variant, 41 with the logopenic variant and 25 with the semantic variant of primary progressive aphasia. We first analysed a large corpus of spoken language and found that the word types preferred by patients with agrammatism tend to have lower frequencies of occurrence than less preferred words. We then conducted a computational simulation to examine the impact of word frequency on lexical information as measured by entropy. We found that strings of words that exclude highly frequent words have a more uniform word distribution, thereby increasing lexical entropy. To test whether the lexical profile of agrammatism results from their inability to produce long sentences, we asked healthy speakers to produce short sentences during the picture description task. We found that, under this constrained condition, a similar lexical profile of agrammatism emerged in the short sentences of healthy individuals, including fewer function words, more nouns than verbs and more heavy verbs than light verbs. This lexical profile of short sentences resulted in their lower average word frequency than unconstrained sentences. We extended this finding by showing that, in general, shorter sentences get packaged with lower-frequency words as a basic property of efficient language production, evident in the language of healthy speakers and all primary progressive aphasia variants.

## Teaser

Rezaii *et al*. provide an information-theoretic account of agrammatism. They show that the lexical profile of agrammatism results from a process that ops for lower-frequency words in response to patients’ deficiency in producing complex sentences. This response that stems from a basic property in language production increases lexical information.

## Graphical Abstract

**Graphical abstract:**
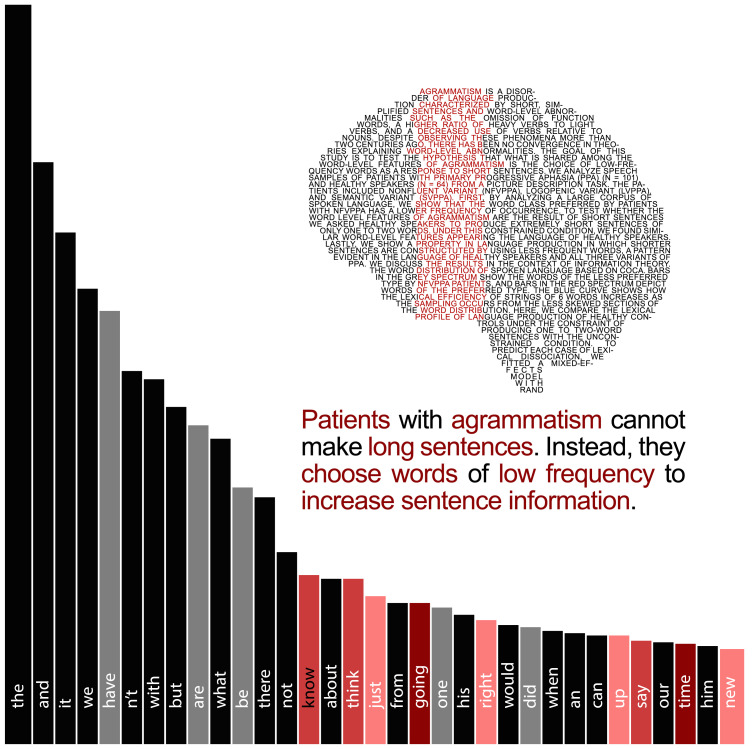

## Introduction

Impaired functioning of the left hemisphere's inferior frontal areas is associated with a distinct style of language production known as agrammatism. Common symptoms of agrammatism include short, simplified sentences, the omission of function words, a decreased use of verbs relative to nouns and increased use of heavy verbs than light verbs.^1–9^ Despite observing some of these symptoms for over two centuries, accounts aiming to explain the underlying mechanism of agrammatism have not converged.

One of the most consistently reported features of agrammatism is the omission of function words, such as pronouns, auxiliary verbs and determiners.^4,5,10–12^ In contrast to content words that carry the main message of a sentence, function words primarily play a grammatical role with likely distinct storage and access processes than those for content words.^13–15^ Because agrammatism is thought to disrupt the syntactic structure of a sentence, access to function words has been hypothesized to get more affected than access to content words.^16–18^ Another key symptom of agrammatism is the increased use of nouns over verbs.^19–26^ Since both nouns and verbs are content words, the dual system for retrieving function/content words cannot explain the noun/verb dissociation. Instead, this verb deficit has been attributed to the greater syntactic complexity of verbs in processing the relationships among sentence elements.^6,21,27–29^ Lastly, the third and more recent case of lexical dissociation in agrammatism is an increased use of heavy verbs relative to light verbs compared to healthy language production.^30,31^ Light verbs such as *go*, *do, get* and *take* are semantically more general and associated with less specific objects.^32–34^ It is suggested that heavy verbs are more resistant to disruption as they are semantically richer and more specific to a particular context than light verbs.^31^ The heavy/light verb distinction represents yet another case of lexical dissociation with a proposed underlying mechanism not matching those for content/function words and noun/verb dissociations.^35^ In the absence of a converging account, agrammatism is mainly conceptualized as a multi-component disorder in which each component results from a distinct mechanism. The co-occurrence of various symptoms is thus ascribed to the proximity of their underlying neural circuitries.^6^

In this article, we introduce an alternative conceptual framework based on information theory that explains the lexical profile of agrammatism not as a conglomerate of independent disorders but as a monolithic compensatory response. According to this framework, the agrammatic lexical profile arises from a process that opts for lower-frequency words in language production to increase sentence information. This strategic choice of words is a response to the agrammatic patients’ core deficit in producing long, complex sentences. We evaluate this hypothesis by analysing the language of patients with primary progressive aphasia (PPA) and healthy speakers as they participate in a picture description task. PPA has three subtypes, with the non-fluent variant (nfvPPA) characterized by agrammatic/effortful speech and two other variants, logopenic (lvPPA) and semantic (svPPA), by lexicosemantic deficits.^36^

First, we test the hypothesis that the three cases of lexical dissociation in agrammatism are the products of choosing low-frequency words. We analyse a large corpus of spoken language consisting of over 115 million words to show that the word types preferred by nfvPPA patients—content over function words, nouns over verbs and heavy over light verbs—indeed occur less frequently than the alternative type. This work will complement previous literature showing function words are more common than content words by extending the analyses to other word types in the spoken modality.^13,37^

Next, we show how choosing low-frequency words increases the lexical information of sentences based on concepts from Shannon's Mathematical Theory of Communication.^38^ The information content of a phenomenon relates to its predictability. Highly predictable phenomena yield little information, and more surprising ones offer more information. Using low-frequency words increases the information content of a sentence in two ways. First, low-frequency words are generally less predictable than high-frequency words in a sentence. For example, the lower-frequency word *Oxalis* is also less predictable than the word *plant* from the context, ‘*In my garden, I grow this type of* ….’. The relationship between the frequency of a word and its predictability in a context is statistically significant^39^ but not absolute because low-frequency words can become more predictable in certain contexts.^40^

The second way in which using low-frequency words enhances the information content of a sentence is by increasing its lexical entropy. Entropy is another way to measure predictability, with phenomena of high entropy being less predictable, hence more informative. The maximum lexical entropy of a sentence occurs when its words have an equal probability of appearance or uniform distribution, for example, when each word appears only once. Conversely, a skewed word distribution decreases lexical entropy, for example, when a word appears more than others. Now the question is how selecting low-frequency words from the lexicon of all possible words would result in higher lexical entropy of sentences. The answer to this question lies in the properties of the word distribution of the lexicon. As famously shown through Zipf's law, the lexicon of all words of a language contains few words with a very high frequency of occurrence and the rest with a much lower frequency.^41,42^ This pattern results in a highly skewed distribution for the high-frequency words and a long uniform tail for the low-frequency words. As a result, we expect selecting words from the more uniformly distributed tail of the lexicon for making sentences to result in higher lexical entropy than choosing from the skewed part of the distribution. We will run a computational simulation to show the statistical relationship between the use of low-frequency words and higher lexical entropy at the sentence level.

Lastly, we test the hypothesis that the lexical profile of agrammatism arises as a response to the central deficit of patients in producing long, complex sentences. We ask healthy individuals to describe the same picture using short sentences of only one to two words. Our expectation is that the constraint on sentence length will induce a similar lexical profile of agrammatism. We then test the general hypothesis that shorter sentences contain lower-frequency words as a basic property of language production in healthy speakers and all three variants of PPA.

## Materials and methods

### Participants

For this cross-sectional study, we recruited 100 patients with PPA from an ongoing longitudinal study conducted in the Frontotemporal Disorders Unit of Massachusetts General Hospital (MGH). Comprehensive clinical and language assessments were used to characterize and subtype patients into nfvPPA (*n* = 34), svPPA (*n* = 25) and lvPPA (*n* = 41), as previously described.^43^ All patients were native English speakers. The number of patients in this cohort reflects the data available when we started this study. We included ratings on the Progressive Aphasia Severity Scale which uses the clinician's best judgement, integrating information from the patient's examination and a companion's description of routine daily functioning.^44^ The Progressive Aphasia Severity Scale includes boxes for fluency, syntax, word retrieval and expression, repetition, auditory comprehension, single-word comprehension, reading, writing and functional communication. The Progressive Aphasia Severity Scale Sum-of-boxes is the sum of each of the box scores. The study also includes 34 age-matched healthy controls enrolled through the Speech and Feeding Disorders Laboratory at the MGH Institute of Health Professions. These participants passed a cognitive screen, were native English speakers, and had no history of neurologic or developmental speech or language disorders. For the constrained language task, we recruited a separate cohort of 31 individuals from Amazon's Mechanical Turk. Amazon's Mechanical Turk participants filled out a short survey about their neurological and language backgrounds. Only language samples from participants who were native English speakers with no self-reported history of brain or speech-language disorder, either developmental or acquired, were included in the analyses. These participants had an average age of 47.6 with an average year of education of 16.1. In this cohort, 21 participants were female, and 27 were right-handed. The clinical and demographic information on the participants is shown in Table 1. All participants provided informed consent following guidelines established by the Mass General Brigham Healthcare System Institutional Review Boards, which govern human subjects research at MGH. The Brain Resilience in Aging: Integrated Neuroscience Studies (BRAINS) at MGH approved data collection for Amazon's Mechanical Turk individuals.

**Table 1:** Clinical and demographic characteristics of participants performing the unconstrained task^a^

	nfvPPA	lvPPA	svPPA	Controls	Statistics (*P*-value)
Sample size	34	41	25	34	
Mean age (SD)	65.87 (9.05)	65.45 (6.92)	60.93 (7.84)	64.84 (8.44)	*F* = 1.384 (0.252)
Handedness, right	87%	80.6%	88.9%	73.3%	*χ*^2^ = 7.080 (0.313)
Mean years of education (SD)	17.31 (7.43)	16.63 (2.22)	16.56 (1.71)	15.87 (1.54)	*F* = 0.622 (0.592)
Female:male	21:13	18:23	15:10	19:15	*χ*^2^ = 2.911 (0.406)
PASS sum-of-boxes	5.70 (3.56)	5.77 (2.63)	5.00 (2.21)		*F* = 0.494 (0.612)
Mean MoCA score (SD)	23.38 (4.34)	20.52 (4.57)	19.60 (6.91)		*F* = 3.101 (0.052)

MoCA, Montreal Cognitive Assessment; PASS, Progressive Aphasia Severity Scale; SD, standard deviation.

a We report *F*-value for ANOVA and *χ*^2^ for chi-square tests.

### Language samples

For the unconstrained condition, the participants were asked to look at a drawing of a family at a picnic from the Western Aphasia Battery–Revised^45^ and describe it using as many complete sentences as they could. Responses were audio-recorded in a quiet room and later transcribed by a researcher blind to the grouping. For the constrained condition, we asked the Amazon's Mechanical Turk participants to describe the same picture using either one or two-word sentences. A sentence was defined as an independent clause and all clauses dependent on it.^46^ As language data were sparse for sentences containing more than 20 words (∼1% of all utterances), the scatter plots show 99% of sentences with a length of ≤20.

### Text analysis of language samples

We used Quantitext, a text analysis toolbox developed in MGH Frontotemporal Disorders Unit, to automatically generate a set of quantitative language metrics to increase the precision and objectivity of language assessments while reducing human labour (along with the goals outlined previously).^47^ The toolbox uses several natural language processing tools, such as Stanza^48^ and text analysis libraries in R. Quantitext receives transcribed language samples as input and generates several metrics, such as sentence length, log word frequency, log syntax frequency,^49^ content units,^50^ the efficiency of lexical and syntactic items,^51^ part of speech tags and the distinction of heavy and light verbs. Nouns, verbs (except for auxiliary verbs), adjectives and adverbs are considered content words, and others as function words. The toolbox classifies the verbs *go, have, do, come, give, get, make, take, be, bring, put* and *move* as light verbs while excluding auxiliaries from this list.^31^ All other verbs are classified as heavy verbs.

### Corpus analysis and measuring word frequency

For corpus analysis and the measurement of word frequency, we used the Corpus of Contemporary American English (COCA).^52^ The corpus comprises 1 billion words of contemporary American English in eight genres: TV/movies, spoken, fiction, magazine, newspaper and academic. To best represent our study cohort, we used the spoken genre of COCA, which consists of transcripts of unscripted conversations from about 150 different TV and radio programmes with 115 937 138 lemmatized and 121 465 711 token words.

### Measuring the lexical entropy normalized by word count

Given a string of words with probabilities *p*(*x*_1_) for the first word type to *p*(*x_n_*) for the *n*th word type, the lexical entropy of the string normalized by word count is calculated by the following formula.

$$-\overset{n}{\underset{i=1}{\sum }}\;p(x_{i})\mathrm{lo}g_{2}(p(x_{i}))/\mathrm{lo}g_{2}(n)$$

Normalized entropy takes values from 0 to 1.

### Statistical analysis

For the statistical analyses of this study, we used the R software version 4.1.2. We used independent *t*-tests to compare the log frequency of the lemmatized words of COCA. We used generalized additive models (GAM) to estimate the smooth but potentially non-linear relationship between sentence length and word frequency. GAM is a generalized linear model in which the mean of the outcome is a sum of unknown smooth univariate functions of continuous predictors. Spline functions are popular for bases in GAM because they can approximate smooth functions when the number of internal knots is large enough.^53^ Splines are piece-wise polynomial functions and the places where two neighbouring pieces of the polynomial meet are known as the internal knots. A spline function becomes more flexible (i.e. capable of describing a wider range of non-linear functions) as the number of internal knots increases. However, too many internal knots usually lead to overfitting, and thus, the number of internal knots should be selected carefully. To avoid overfitting and increase the generalizability of the fitted model, a smoothness penalty on the spline function is usually employed to prevent the model from interpolating. A commonly used class of penalties targets the L2 norm of the derivative of a given order and controls the complexity of the fitted GAM. We use thin plate regression splines^54^ as the basis functions and set the number of internal knots of the spline GAM model shows the degree of curvature of the relationship. The value of effective degrees of freedom (EDF) formed by the GAM model shows the degree of curvature of the relationship. A value of 1 for EDF is translated as a linear relationship. Values larger than one denote a more complex relationship between the predicting and outcome variables. We used the *gam* function in the *mgcv* package in R to fit the model.^55^ We included in the model separate spline functions of sentence length for each group of subjects (e.g. PPA variants versus healthy controls) and a subject-specific random slope. The model parameters were estimated via the restricted maximum likelihood method.^56^ To test whether the relationship between word frequency and sentence length was different in PPA variants compared to healthy controls, we performed a generalized likelihood ratio test for penalized splines.^57^ To compare the features of agrammatism across different groups, we used mixed-effects models with subject-specific random intercept via the lme4 package in R.^58^

## Results

### Analysing a large corpus of spoken data

**Figure 1:**
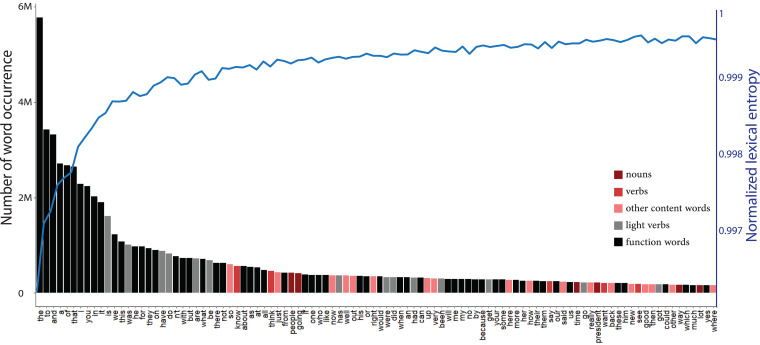
**Words of the type preferred by patients with nfvPPA and the impact of this choice on lexical entropy**. The rank-ordered bar graph shows the word distribution of spoken language based on COCA. Bars in the grey spectrum show the words of the less preferred type by nfvPPA patients, and bars in the red spectrum depict words of the preferred type. The blue curve shows how the normalized lexical entropy of six-word strings increases as the sampling occurs from the less skewed sections of the distribution.

Here, we analysed a large corpus of spoken language from COCA. Using independent *t*-tests, we found that in spoken language, content words have a lower log frequency (mean = 3.44, SD = 2.32) than function words (mean = 5.70, SD = 3.56) [*t*(913.07) = −19.049 *P* < 0.001] consistent with the previous literature. Furthermore, we found that nouns, on average, have a lower log frequency of occurrence (mean = 3.58, SD = 2.34) than verbs (mean = 4.68, SD = 2.30) [*t*(8728.8) =−32.774, *P* < 0.001]. Similarly, heavy verbs showed a lower log frequency (mean = 4.67, SD = 2.27) than light verbs (mean = 12.84, SD = 1.47) [*t*(11.11) = −19.235, *P* < 0.001] in the spoken corpus. As such, the word types preferred by patients with nfvPPA have a lower frequency of occurrence than the less preferred words. Figure 1 shows the word distribution of COCA with colour-coded bars. Words of the type preferred by patients with agrammatism, bars in the red spectrum, are mainly located in the low-frequency tail of the distribution, while words of the less preferred type, bars in the grey spectrum, are in the skewed high-frequency part of the distribution.

### A computational simulation to show that making sentences from the low-frequency tail of the lexicon results in higher lexical entropy

Here, we use a computational simulation to show that excluding higher-frequency words in a sentence increases normalized lexical entropy. In this simulation, we created strings of six words, the average sentence length produced by patients with nfvPPA in our cohort. First, we created a set of 10 000 strings with a length of six words by randomly sampling from the entire COCA, obeying the frequency weights of the corpus. This first set is the baseline, and the rest will be test sets. For the second set, we created another 10 000 six-word strings, excluding the most frequent word of the lexicon, ‘the’, from sampling. For the third set, we excluded the two most frequent words from sampling. We followed this procedure to create 100 sets of 10 000 strings of six words and calculated the normalized lexical entropy of each set.

Interestingly, we found that by only excluding ‘the’ from sampling, the normalized lexical entropy of sentences significantly increased (*t* = 5.2178, *P* < 0.001). The difference became larger as more high-frequency words were excluded from sampling. The blue curve in Fig. 1 shows the increase in normalized lexical entropy as a function of sampling from the lower-frequency sections of the lexicon. Supplementary material shows the statistical significance of the difference between the normalized lexical entropy of the baseline set and each of the 99 test sets. We replicated these findings for strings with a length varying from 2 to 15, as shown in Supplementary Fig. 1 and Table 1.

### Analysing the effect of length on the lexical profile of a sentence

#### Comparing language production of healthy individuals under the unconstrained and sentence length-constrained conditions

Here, we compare the lexical profile of sentences of healthy individuals under the constraint of producing one- to two-word sentences with the unconstrained condition. To predict each case of lexical dissociation, we fitted a mixed-effects model with random effects for subjects with the condition of language production as a predictor. We found that the constrained sentences of healthy individuals contained a higher proportion of content words to all words (mean = 0.91, SD = 0.26) than the unconstrained condition (mean = 0.45, SD = 0.12) (*β* = 0.460, SE = 0.014, *t* = 33.34, *P* < 0.001). The proportion of nouns to nouns plus verbs was higher in the constrained (mean = 0.83, SD = 0.32) than unconstrained condition (mean = 0.61, SD = 0.24) (*β* = 0.218, SE = 0.030, *t* = 7.24, *P* < 0.001). Lastly, constrained sentences had a higher proportion of heavy verbs to all verbs (mean = 0.94, SD = 0.24) than the unconstrained condition (mean = 0.63, SD = 0.42) (*β* = 0.308, SE = 0.046, *t* = 6.702, *P* < 0.001). Interestingly, constrained sentences had more verbs in *-ing* form (mean = 0.80, SD = 0.40) than unconstrained sentences (mean = 0.44, SD = 0.43) (*β* = 0.308, SE = 0.068, *t* = 4.506, *P* < 0.001).

**Figure 2:**
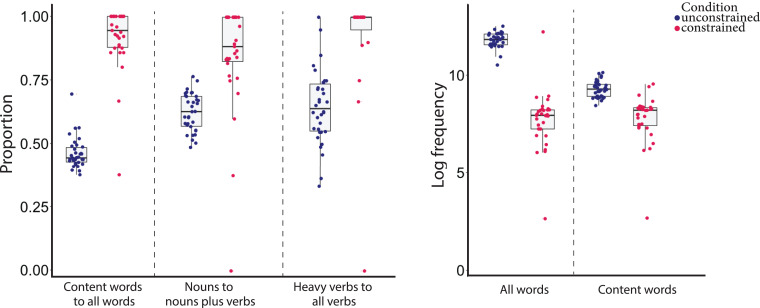
**Box plots of the lexical profile of sentences under unconstrained and constrained conditions (*n* = 65).** Boxes show the 25th, 50th and 75th percentile, and the whiskers represent the minimum and maximum values, excluding outliers.

Furthermore, in a mixed-effects model with random effects for subjects with the condition of language production and sentence length as predictors, the log frequency of all words was lower in the constrained (mean = 8.03, SD = 2.10) than unconstrained condition (mean = 11.86, SD = 0.89) (*β* = −3.508, SE = 0.171, *t* = −20.541, *P* < 0.001). The log frequency of content words of a given sentence was also lower in the constrained (mean = 8.13, 1.94) than unconstrained condition (mean = 9.36, SD = 1.38) (*β* = −0.960, SE = 0.179, *t* = −5.36, *P* < 0.001). Figure 2 shows the lexical profile of the sentences of healthy speakers under the two conditions.

#### Evaluating sentence length–word frequency relationship in healthy individuals

**Figure 3:**
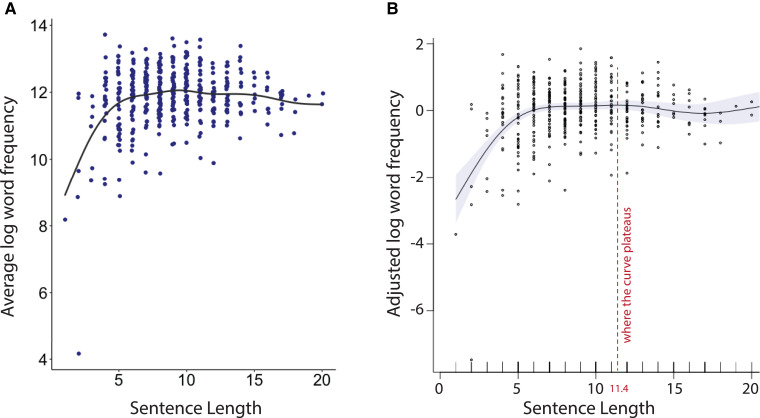
**Sentence length–word frequency relationship in the healthy control group (*n* = 34).** (**A**) The scatter plot shows the relationship between the average log frequency of words within a given sentence and the sentence length in the unconstrained language of healthy individuals. (**B**) The GAM partial effect plot of the smooth term for word frequency is used to determine where the curve begins to plateau (dashed line). The shaded areas indicate the pointwise 95% confidence intervals of the fitted curves.

We tested the general hypothesis that shorter sentences contain lower-frequency words in the language production of healthy speakers. We fitted a GAM to the sentences of healthy speakers in the unconstrained condition with a subject-specific random intercept to model the relationship between sentence length and word frequency. We found that sentence length could predict the average log frequency of all words within that sentence (EDF = 6.97, *P* < 0.001). As can be seen in Fig. 3A, the sentence length–word frequency relationship is approximately linear until the curve starts to plateau. To determine the sentence length where the curve plateaus, we created a random data set where the value of sentence length varied from 1 to 20 with 0.1 increments. We then used the fitted GAM of sentence length–word frequency in the unconstrained healthy language production data to predict the word frequency of the randomly created data set. We found that the maximum word frequency occurs at a sentence length of 11.4 (Fig. 3B).

Since longer sentences tend to contain more function words, we re-examined the sentence length–word frequency relationship after adding the proportion of function words to all words to the statistical model. We used a multivariable GAM to predict the average frequency of all words of a sentence from its length and the proportion of function words to all words with a subject-specific random intercept. We continued to find a significant relationship between sentence length and the average log frequency of all words (EDF = 5.12, *P* < 0.001) and the proportion of function words to all words (EDF = 2.90, *P* < 0.001). Furthermore, we evaluated the relationship between sentence length and the average log frequency of the content words within a sentence in a similar GAM model as that for the average log frequency of all words. We found that sentence length could predict the average log frequency of content words within a sentence (EDF = 4.05, *P* = 0.002).

### Lexical information across the four groups

First, we compare the average log frequency of words and the normalized lexical entropy of sentences among PPA variants and healthy controls. We fitted a mixed-effects model with random effects for subjects to predict the average log frequency of all words with group and sentence length as predictors. We found that patients with nfvPPA produce sentences with a lower average log frequency of all words (mean = 10.88, SD = 1.92) than that of healthy controls (mean = 11.86, SD = 0.89) (*β* = −0.659, SE = 0.164, *t* = −4.015, *P* < 0.001), lvPPA (mean = 11.47, SD = 1.53) (*β* = −0.435, SE = 0.152, *t* = −2.845, *P* = 0.005) and svPPA (mean = 11.60, SD = 1.40) (*β* = −0.540, SE = 0.172, *t* = −3.131, *P* < 0.001). We ran a similar model by adding the proportion of function words to all words as a third predictor and continued to find that patients with nfvPPA produce sentences with a lower log frequency of all words than that of healthy controls (*β* = −0.572, SE = 0.149, *t* = −3.842, *P* < 0.001), lvPPA (*β* = −0.280, SE = 0.138, *t* = −2.047, *P* = 0.042) and svPPA (*β* = −0.311, SE = 0.157, *t* = −1.981, *P* = 0.049). Furthermore, we fitted a mixed-effects model with random effects for subjects to predict the average log frequency of content words with group and sentence length as predictors. We found that patients with nfvPPA produce sentences with a lower log frequency of content words (mean = 8.40, SD = 1.84) than those of healthy controls (mean = 9.36, SD = 1.38) (*β* = −0.700, SE = 0.180, *t* = −3.899, *P* < 0.001), lvPPA (mean = 9.81, SD = 1.99) (*β* = −0.124, SE = 0.166, *t* = −7.467, *P* = 0.040) and svPPA (mean = 10.09, SD = 1.68) (*β* = −0.147, SE = 0.187, *t* = −7.820, *P* < 0.001).

To compare normalized lexical entropy, we fitted a mixed-effects model with random effects for subjects to predict normalized lexical entropy with group as a predictor. We found that patients with nfvPPA produce sentences with higher normalized lexical entropy (mean = 0.995, SD = 0.11) than controls (mean = 0.993, SD = 0.01) (*β* = 0.003, SE = 0.001, *t* = 2.251, *P* = 0.026), lvPPA (mean = 0.993, SD = 0.01) (*β* = 0.002, SE = 0.001, *t* = 2.100, *P* = 0.038) but not different from svPPA (mean = 0.994, SD = 0.01) (*β* < 0.001, SE = 0.001, *t* = 0.684, *P* = 0.495).

Lastly, in a mixed-effects model with random effects for subjects to predict entropy from word frequency, we found a significant negative relationship between the two, showing sentences with lower-frequency words have higher lexical entropy (*β* = 0.001, SE < 0.001, *t* = −3.894, *P* < 0.001).

#### Comparing sentence length–word frequency relationships among healthy speakers and three PPA variants

**Figure 4:**
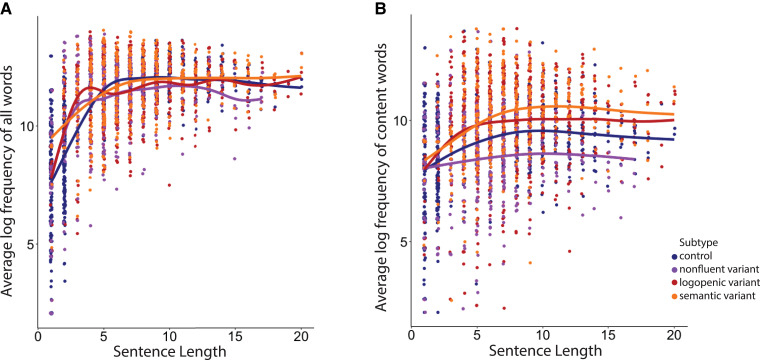
**Sentence length and word frequency relationship across four groups (*n* = 165).** (**A**) The scatterplot shows the relationship between sentence length and the average log frequency of all words within a given sentence in all groups. (**B**) The scatterplot shows the relationship between sentence length and the average log frequency of content words within a given sentence in all groups combined.

Here, we evaluate the relationship between the log word frequency of all words in a given sentence and sentence length in the three variants of PPA and compare it with that of healthy controls. We fitted a GAM to sentences produced by all groups with a subject-specific random intercept to model the relationship between sentence length and word frequency. The results showed that the average log frequency of all words could be predicted from sentence length (EDF = 7.54, *P* < 0.001) (Fig. 4A). To examine whether there was an interaction between word frequency–sentence length relationship and group, we performed a generalized likelihood ratio test for penalized splines to examine. We found no interaction between the average log word frequency–sentence length curve and group (d.f. = −9.247, Deviance = 14.03, *P* = 0.927), showing that the curves have a similar overall shape across four groups.

We repeated these analyses for content words and found that the average log frequency of content words could be predicted from sentence length (EDF = 5.75, *P* < 0.001) (Fig. 3B). Similarly, we found no interaction between the shape of sentence length–content word frequency curve and group (d.f. = −8.20, Deviance = −29.25, *P* = 0.265).

## Discussion

In this study, we provided a parsimonious account of agrammatism based on information theory. We showed that the particular lexical profile of agrammatism arises from a process that favours low-frequency words in response to patients’ core difficulty in producing long, complex sentences. Previous research has shown that speakers of a language are sensitive to the probability distribution of words and syntactic rules of that language.^59–61^ This sensitivity to the statistical properties of language allows learners, including infants, to discover words, syntactic structures and sound patterns from the ground up.^62^ Here, we show that sensitivity to word frequency can further assist patients with agrammatism in optimizing the lexical information of their short sentences. While sentences generated from this cognitive process may appear disjointed, they capture the essence of the intended meaning. Furthermore, as an integral part of our proposal, we provided a computational simulation to show how selecting low-frequency words increases the lexical entropy of sentences. We found that even excluding the single most frequent word of the lexicon, ‘*the*’, increases the lexical entropy of the resulting word strings. Our simulation further delineated the relationship between word frequency and lexical entropy. Although the average word frequency and lexical entropy of sentences are two related measures of predictability, we found word frequency to be a more specific index of lexical information for clinical purposes as it better differentiated PPA variants.

Our work, initially outlined in Rezaii *et al*.,^63^ revives a series of accounts from the past century based on compensation. According to ideas regarding the ‘economy of effort’, the intensive effort required to articulate speech forces non-fluent patients to plan short strings of only essential words that exclude function words.^64,65^ Despite its plausibility, this idea did not fare well during the pre-information theory era, likely because it lacked a robust way to measure information. Our study operationalizes the measuring of lexical information and extends the compensation idea to other cases of lexical dissociation in agrammatism beyond just the function/content word dissociation. The theoretical foundation of this proposal was further supported by a review paper that regarded the compensatory response of patients with agrammatism as a rational behaviour in the face of their increased cost of language production.^66^

The conceptual framework of this study shifts away from the syntax-centric accounts that consider a deficit in syntax processing to be the cause of the lexical profile of agrammatism. Under such accounts, it remained unclear why patients with agrammatism could have intact online access to the verb lexicon,^67,68^ access to all possible argument structures of verbs during online sentence processing,^67,68^ and minimal errors in using function words in sentence completion tasks.^69^ Unlike syntax-centric accounts, these results, which indicate near-normal lexical production in agrammatism, fit well within the information-theoretic account of the disorder.

Our study further showed that the lexical compensation strategy in agrammatism is not unique to patients with nfvPPA but rather highlights a fundamental property of normal language production. When healthy speakers were constrained to produce one- and two-word sentences, their language exhibited features similar to those of patients with agrammatism, including an increase in the proportion of content words to all words, nouns to verbs and heavy verbs to light verbs. In a constrained production, it is logical to expect function words and light verbs to be dropped due to their semantic emptiness. However, the relationship between the increased use of nouns and information compression may not be readily apparent. Previous research has shown that in writing, particularly in scientific abstracts with strict word limits, the ratio of nouns to verbs tends to increase.^70–72^ The information compression capacity of nouns stems from several of their inherent properties.^73–76^ Nominalization involves transferring the information content from a clause to a noun phrase, which can lead to shorter and simpler sentences. This process also allows for stacking groups of meaning within noun phrases, which is a more economical approach compared to stacking clauses, as the latter can result in verbosity and complexity. Moreover, using noun phrases in place of clauses anonymizes the agent of the action and shifts the focus from the actor to the action while saving words.

Also similar to patients with agrammatism,^77–80^ the constrained language of healthy speakers resulted in more verbs in *-ing* form. For example, in describing a man who was fishing on a pier, both groups used the sentence ‘*man fishing*’. Deviations in verb inflection in agrammatism reflect a complex phenomenon involving multiple factors, such as the type of production task and the cognitive load associated with it, the accessibility of certain verb forms and the verb properties specific to a language.^81–83^ Our findings on the increased use of participles under production constraints suggest that attempts to optimize information transfer could be another determining factor in verb morphology. Using participles without auxiliaries might offer a concise way of expressing an action while emphasizing its progressive aspect.

Following the observation of the lexical profile of the length-constrained condition, we tested a general relationship between the average word frequency and the length of a sentence. We found that up to a sentence length of ∼11 words, shorter sentences predict lower-frequency words. The sentence length–word frequency relationship showed to be a fundamental property of language production that is preserved even in patients with lexicosemantic deficits. This fundamental property can be explained by forces that shape human language to transfer the maximum amount of information with the least effort.^84^ When speakers plan to communicate a particular idea, they have multiple options regarding the choice of words, syntax and sentence length.^85,86^ However, some formulations may be inefficient due to redundancy or verbosity and are therefore avoided. Constructing longer sentences^87–91^ and retrieving lower-frequency words^92–94^ are two cognitively demanding processes that share the same goal of conveying more information. Therefore, balancing the two sources of information would be an efficient way of communicating a message. This efficiency of communication draws on essential properties of language production. One property is the existence of a self-monitoring system that tracks how well the message of a sentence is conveyed.^95^ The other property is the close interactivity between the lexical and structural sources of information.^35,96,97^ This interactivity enables patients with sentence structure deficits to compensate by using low-frequency words and patients with lexicosemantic impairments by choosing more complex structures to convey their message.^98^

From this work, we cannot determine the functional locus of bottleneck^99^ in producing long, complex sentences in patients with nfvPPA. Among alternative candidates are impairments at the conceptualization and language planning stages, poor executive function,^100^ impaired working memory, deficient phonological processing^23,101^ and motor planning deficits.^65^ Future work is needed to elucidate the core mechanism underlying the fundamental limitation on sentence production in patients with ‘agrammatism’.

## Supplementary Material

fcad136_Supplementary_Data

## Data Availability

The language scores of anonymized patients and healthy individuals and the R code used for data analysis are available at the following link. https://dataverse.harvard.edu/dataset.xhtml? persistentId=doi:10.7910/DVN/SOMVGP.

## Abbreviations

COCA =: Corpus of Contemporary American English
EDF =: effective degrees of freedom
GAM =: generalized additive models
lvPPA =: logopenic variant primary progressive aphasia
MGH = =: Massachusetts General Hospital
nfvPPA = =: non-fluent variant primary progressive aphasia
PASS =: Progressive Aphasia Severity Scale
PPA =: primary progressive aphasia
svPPA =: semantic variant primary progressive aphasia
