# Supplementary material for: Less is more in language production: an information-theoretic analysis of agrammatism in primary progressive aphasia
Source: Brain Commun. 2023 Apr 25;5(3):fcad136. doi: 10.1093/braincomms/fcad136 (PMC10263269; doi:10.1093/braincomms/fcad136)
Supplement: fcad136_Supplementary_Data [file fcad136_Supplementary_Data.zip › Supplementary Material.docx]

**Supplementary material**

Please refer to Section 2 of the main manuscript for details about the simulation. In Supplementary Table 1, the *word rank* and *word* columns show the point in the word distribution based on COCA, where the sampling to create word strings starts. For example, the first row shows sampling begins from “the”, including the entire word distribution. The outcome constitutes the baseline set; the rest will be test sets. The second row shows sampling begins from “to”, excluding “the” and including all other words. In the third row, sampling starts from “and”, excluding “the” and “to”. We continue this process for one hundred sets. The *mean* columns show the average normalized lexical entropy of 10,000 strings of words for each string length (Supplementary Figure 1). The *t.statictics* and *p.values* show the difference between the normalized lexical entropy of the test sets and the baseline set for each string length.


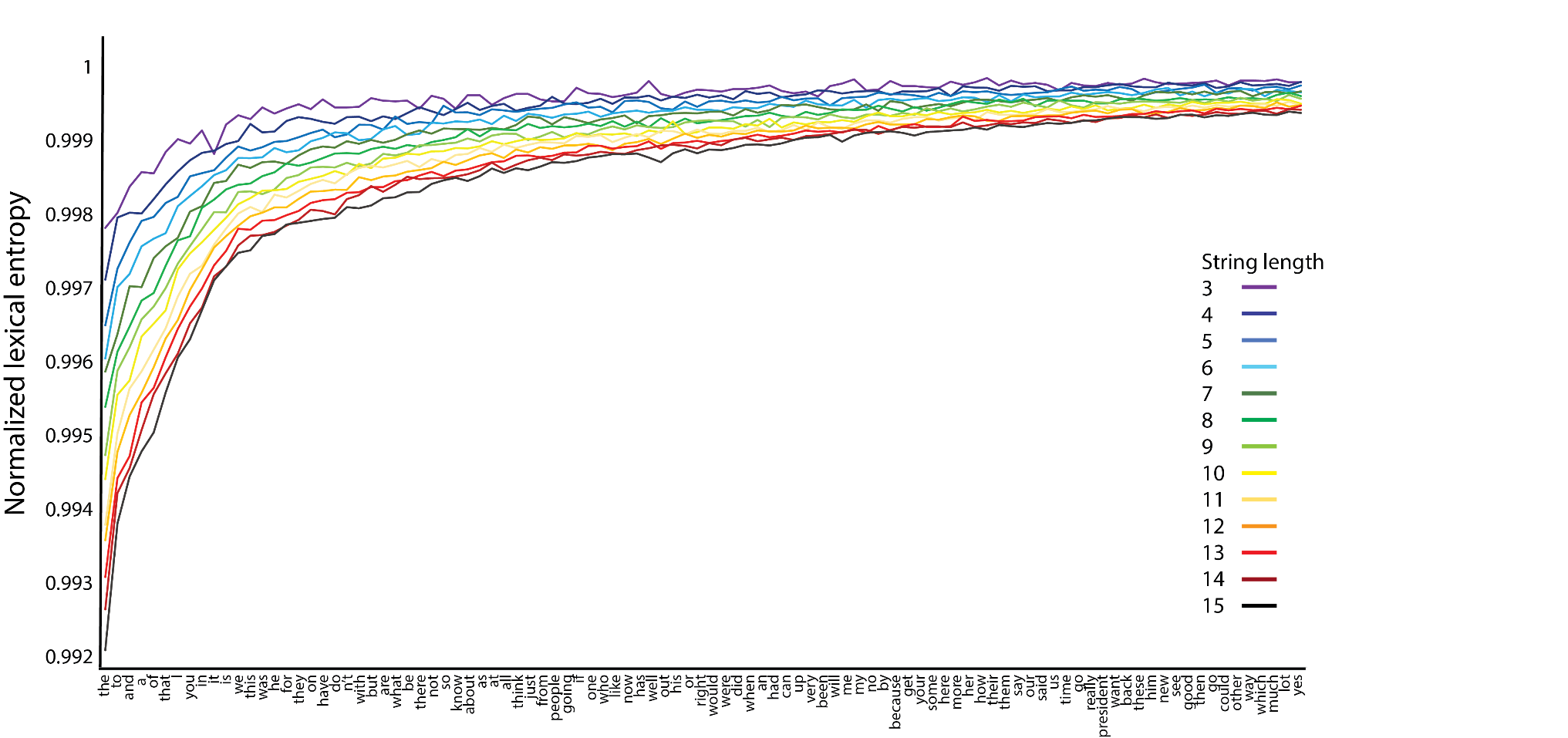


**Supplementary Figure 1** shows the increase in normalized lexical entropy when less-frequent words of the distribution are used to make strings of lengths varying from 2 to 15 words.
