# Supplementary material for: Less is more in language production: an information-theoretic analysis of agrammatism in primary progressive aphasia
Source: Brain Commun. 2023 Apr 25;5(3):fcad136. doi: 10.1093/braincomms/fcad136 (PMC10263269; doi:10.1093/braincomms/fcad136)
Supplement: fcad136_Supplementary_Data [file fcad136_Supplementary_Data.zip › Supplementary Table 1.pdf]

Supplementary Table 1. The normalized lexical entropy of the baseline set (red highlights) and test sets (blue highlights) and the statistical difference between them. The procedure was repeated for string lengths of 3 to 15 words.

Word Rank	Word	mean-Length 03	t.stat-Length 03	p.value-Length 03	mean-Length 04	t.stat-Length 04	p.value-Length 04	mean-Length 05	t.stat-Length 05	p.value-Length 05	mean-Length 06	t.stat-Length 06	p.value-Length 06	mean-Length 07
1	the	0.9979	NA	NA	0.9972	NA	NA	0.9966	NA	NA	0.9961	NA	NA	0.996
2	to	0.9981	1.66	0.0977	0.998	4.84	<0.001	0.9973	2.68	0.007	0.9971	5.22	<0.001	0.9965
3	and	0.9984	3.58	<0.001	0.9981	5.26	<0.001	0.9977	5.06	<0.001	0.9973	6.42	<0.001	0.9971
4	a	0.9986	4.44	<0.001	0.9981	5.14	<0.001	0.998	7.16	<0.001	0.9976	9.33	<0.001	0.9971
5	of	0.9986	4.36	<0.001	0.9983	6.46	<0.001	0.998	7.57	<0.001	0.9977	10.08	<0.001	0.9975
6	that	0.9989	5.66	<0.001	0.9985	7.82	<0.001	0.9982	9.02	<0.001	0.9978	10.73	<0.001	0.9976
7	i	0.9991	6.47	<0.001	0.9986	9.14	<0.001	0.9983	9.65	<0.001	0.9982	13.67	<0.001	0.9977
8	you	0.999	5.73	<0.001	0.9988	10.26	<0.001	0.9986	11.89	<0.001	0.9983	14.82	<0.001	0.9981
9	in	0.9992	7.02	<0.001	0.9989	11.09	<0.001	0.9986	12.27	<0.001	0.9984	15.94	<0.001	0.9982
10	it	0.9989	5.14	<0.001	0.9989	11.3	<0.001	0.9987	12.57	<0.001	0.9986	17.39	<0.001	0.9985
11	is	0.9993	6.84	<0.001	0.999	11.9	<0.001	0.9988	13.88	<0.001	0.9987	17.96	<0.001	0.9985
12	we	0.9994	8.03	<0.001	0.999	12.29	<0.001	0.999	15.32	<0.001	0.9988	19.39	<0.001	0.9987
13	this	0.9993	7.78	<0.001	0.9993	14.11	<0.001	0.9989	14.93	<0.001	0.9988	19.52	<0.001	0.9987
14	was	0.9995	8.56	<0.001	0.9992	13.32	<0.001	0.999	15.39	<0.001	0.9988	19.62	<0.001	0.9988
15	he	0.9994	8.15	<0.001	0.9992	13.41	<0.001	0.999	15.99	<0.001	0.9989	20.74	<0.001	0.9988
16	for	0.9995	8.48	<0.001	0.9993	14.55	<0.001	0.999	16.14	<0.001	0.9989	20.16	<0.001	0.9987
17	they	0.9995	8.77	<0.001	0.9994	15.09	<0.001	0.9991	16.49	<0.001	0.9989	20.51	<0.001	0.9989
18	on	0.9995	8.44	<0.001	0.9993	14.9	<0.001	0.9992	17.17	<0.001	0.999	21.88	<0.001	0.9989
19	have	0.9996	9.11	<0.001	0.9993	14.45	<0.001	0.9992	17.66	<0.001	0.9991	22.25	<0.001	0.999
20	do	0.9995	8.56	<0.001	0.9993	14.22	<0.001	0.9991	16.62	<0.001	0.9992	22.99	<0.001	0.999
21	n't	0.9995	8.56	<0.001	0.9994	15.09	<0.001	0.9991	16.98	<0.001	0.9991	22.91	<0.001	0.999
22	with	0.9995	8.6	<0.001	0.9994	15.14	<0.001	0.9993	18.22	<0.001	0.9991	21.8	<0.001	0.999
23	but	0.9996	9.2	<0.001	0.9993	14.61	<0.001	0.9993	18.28	<0.001	0.9991	22.1	<0.001	0.9991
24	are	0.9996	9.03	<0.001	0.9994	15.18	<0.001	0.9992	17.53	<0.001	0.9992	23.37	<0.001	0.999
25	what	0.9996	8.98	<0.001	0.9993	14.78	<0.001	0.9994	19.41	<0.001	0.9992	23.99	<0.001	0.9991
26	be	0.9996	9.03	<0.001	0.9994	15.57	<0.001	0.9993	18.34	<0.001	0.9991	22.69	<0.001	0.9991
27	there	0.9995	7.8	<0.001	0.9995	16.31	<0.001	0.9993	18.66	<0.001	0.9991	22.68	<0.001	0.9992
28	not	0.9996	9.37	<0.001	0.9994	15.82	<0.001	0.9993	18.51	<0.001	0.9993	24.45	<0.001	0.9991
29	so	0.9996	9.15	<0.001	0.9994	15.26	<0.001	0.9995	20.9	<0.001	0.9993	24.34	<0.001	0.9992
30	know	0.9995	7.8	<0.001	0.9995	16.06	<0.001	0.9994	19.96	<0.001	0.9993	24.63	<0.001	0.9992
31	about	0.9997	9.41	<0.001	0.9996	16.87	<0.001	0.9994	19.97	<0.001	0.9993	24.42	<0.001	0.9992
32	as	0.9997	9.41	<0.001	0.9995	15.96	<0.001	0.9994	19.61	<0.001	0.9993	24.78	<0.001	0.9992
33	at	0.9995	8.73	<0.001	0.9995	16.46	<0.001	0.9995	20.31	<0.001	0.9993	24.18	<0.001	0.9992
34	all	0.9996	9.2	<0.001	0.9995	16.63	<0.001	0.9995	20.15	<0.001	0.9994	25.45	<0.001	0.9992
35	think	0.9997	9.5	<0.001	0.9994	15.87	<0.001	0.9994	19.95	<0.001	0.9993	24.69	<0.001	0.9993
36	just	0.9997	9.5	<0.001	0.9995	16.13	<0.001	0.9995	20.13	<0.001	0.9994	25.66	<0.001	0.9994
37	from	0.9996	9.11	<0.001	0.9995	16.38	<0.001	0.9995	20.39	<0.001	0.9994	25.45	<0.001	0.9994
38	people	0.9996	8.98	<0.001	0.9996	17.49	<0.001	0.9995	20.51	<0.001	0.9994	25.14	<0.001	0.9993
39	going	0.9996	8.98	<0.001	0.9995	16.76	<0.001	0.9995	20.9	<0.001	0.9994	25.56	<0.001	0.9994
40	if	0.9998	9.93	<0.001	0.9995	16.71	<0.001	0.9996	21.29	<0.001	0.9994	25.66	<0.001	0.9993
41	one	0.9997	9.54	<0.001	0.9996	16.87	<0.001	0.9995	20.61	<0.001	0.9994	25.91	<0.001	0.9993
42	who	0.9997	9.5	<0.001	0.9996	17.38	<0.001	0.9995	20.74	<0.001	0.9994	25.29	<0.001	0.9993
43	like	0.9996	9.28	<0.001	0.9995	16.63	<0.001	0.9994	19.91	<0.001	0.9994	25.78	<0.001	0.9994
44	now	0.9997	9.41	<0.001	0.9996	17.53	<0.001	0.9996	21.57	<0.001	0.9994	26.01	<0.001	0.9994
45	has	0.9997	9.58	<0.001	0.9996	17.39	<0.001	0.9996	21.65	<0.001	0.9994	26.11	<0.001	0.9993
46	well	0.9998	10.41	<0.001	0.9997	17.79	<0.001	0.9996	21.44	<0.001	0.9994	25.88	<0.001	0.9994
47	out	0.9997	9.5	<0.001	0.9996	17.17	<0.001	0.9995	20.53	<0.001	0.9994	26.16	<0.001	0.9994
48	his	0.9996	9.28	<0.001	0.9996	17.64	<0.001	0.9995	20.37	<0.001	0.9995	26.12	<0.001	0.9994
49	she	0.9997	9.58	<0.001	0.9996	17.48	<0.001	0.9996	21.48	<0.001	0.9995	26.67	<0.001	0.9994
50	or	0.9997	9.8	<0.001	0.9997	17.9	<0.001	0.9995	20.67	<0.001	0.9995	26.26	<0.001	0.9994
51	right	0.9997	9.75	<0.001	0.9996	17.53	<0.001	0.9996	21.53	<0.001	0.9995	26.04	<0.001	0.9994
52	would	0.9997	9.67	<0.001	0.9997	17.7	<0.001	0.9996	21.54	<0.001	0.9994	25.93	<0.001	0.9994
53	were	0.9997	9.84	<0.001	0.9996	17.33	<0.001	0.9996	21.36	<0.001	0.9995	26.68	<0.001	0.9994
54	did	0.9997	9.84	<0.001	0.9997	18.38	<0.001	0.9996	21.65	<0.001	0.9995	26.46	<0.001	0.9995

55	when	0.9998	9.93	<0.001	0.9997	18.01	<0.001	0.9996	21.36	<0.001	0.9995	26.4	<0.001	0.9994
56	an	0.9998	10.1	<0.001	0.9997	18.22	<0.001	0.9996	21.48	<0.001	0.9995	27.2	<0.001	0.9995
57	had	0.9997	9.71	<0.001	0.9996	17.44	<0.001	0.9996	22.16	<0.001	0.9995	26.87	<0.001	0.9995
58	can	0.9997	9.54	<0.001	0.9997	17.79	<0.001	0.9996	21.69	<0.001	0.9995	26.74	<0.001	0.9995
59	up	0.9997	9.67	<0.001	0.9997	17.95	<0.001	0.9996	21.84	<0.001	0.9996	27.57	<0.001	0.9995
60	very	0.9996	9.32	<0.001	0.9997	18.48	<0.001	0.9996	21.95	<0.001	0.9995	27.06	<0.001	0.9995
61	been	0.9998	10.06	<0.001	0.9997	18.38	<0.001	0.9995	20.84	<0.001	0.9995	27.01	<0.001	0.9995
62	will	0.9998	10.24	<0.001	0.9997	18.06	<0.001	0.9996	21.99	<0.001	0.9995	26.93	<0.001	0.9995
63	me	0.9999	10.55	<0.001	0.9997	18.17	<0.001	0.9996	22.12	<0.001	0.9996	27.72	<0.001	0.9995
64	my	0.9997	9.75	<0.001	0.9997	18.38	<0.001	0.9996	22.2	<0.001	0.9995	26.62	<0.001	0.9995
65	no	0.9997	9.8	<0.001	0.9997	18.38	<0.001	0.9997	22.81	<0.001	0.9996	27.74	<0.001	0.9994
66	by	0.9998	10.41	<0.001	0.9997	17.9	<0.001	0.9997	22.59	<0.001	0.9996	27.97	<0.001	0.9996
67	because	0.9998	10.06	<0.001	0.9997	18.22	<0.001	0.9997	22.47	<0.001	0.9996	27.71	<0.001	0.9996
68	get	0.9998	10.06	<0.001	0.9997	18.32	<0.001	0.9997	22.37	<0.001	0.9996	27.9	<0.001	0.9995
69	your	0.9998	9.97	<0.001	0.9997	18.38	<0.001	0.9996	22.12	<0.001	0.9996	28.05	<0.001	0.9995
70	some	0.9998	9.93	<0.001	0.9998	18.65	<0.001	0.9997	22.76	<0.001	0.9996	27.61	<0.001	0.9995
71	here	0.9998	10.37	<0.001	0.9996	17.74	<0.001	0.9996	22.29	<0.001	0.9996	27.74	<0.001	0.9995
72	more	0.9998	10.15	<0.001	0.9998	19.03	<0.001	0.9997	22.98	<0.001	0.9996	28.17	<0.001	0.9996
73	her	0.9998	10.32	<0.001	0.9998	18.86	<0.001	0.9997	22.85	<0.001	0.9996	28.12	<0.001	0.9996
74	how	0.9999	10.64	<0.001	0.9998	18.7	<0.001	0.9997	22.98	<0.001	0.9996	27.49	<0.001	0.9996
75	their	0.9998	10.1	<0.001	0.9997	18.43	<0.001	0.9997	22.63	<0.001	0.9997	28.52	<0.001	0.9996
76	them	0.9999	10.46	<0.001	0.9998	19.08	<0.001	0.9997	22.34	<0.001	0.9996	27.48	<0.001	0.9996
77	say	0.9998	10.19	<0.001	0.9998	18.97	<0.001	0.9997	22.52	<0.001	0.9997	28.59	<0.001	0.9996
78	our	0.9998	10.28	<0.001	0.9998	19.03	<0.001	0.9997	22.76	<0.001	0.9996	28.17	<0.001	0.9996
79	said	0.9998	10.15	<0.001	0.9997	18.38	<0.001	0.9997	22.66	<0.001	0.9996	28.22	<0.001	0.9996
80	us	0.9997	8.94	<0.001	0.9997	18.48	<0.001	0.9997	23.29	<0.001	0.9996	28.31	<0.001	0.9996
81	time	0.9998	10.28	<0.001	0.9997	18.48	<0.001	0.9998	23.73	<0.001	0.9997	28.89	<0.001	0.9996
82	go	0.9998	10.1	<0.001	0.9998	18.92	<0.001	0.9997	22.99	<0.001	0.9997	28.5	<0.001	0.9997
83	really	0.9998	10.06	<0.001	0.9998	18.92	<0.001	0.9997	23.24	<0.001	0.9997	28.75	<0.001	0.9996
84	presider	0.9998	10.28	<0.001	0.9998	19.19	<0.001	0.9997	23.15	<0.001	0.9997	29.01	<0.001	0.9997
85	want	0.9998	10.15	<0.001	0.9998	19.36	<0.001	0.9998	23.64	<0.001	0.9997	28.76	<0.001	0.9996
86	back	0.9998	10.24	<0.001	0.9998	19.03	<0.001	0.9997	23.33	<0.001	0.9997	28.52	<0.001	0.9996
87	these	0.9999	10.59	<0.001	0.9998	18.92	<0.001	0.9997	22.3	<0.001	0.9997	28.87	<0.001	0.9997
88	him	0.9998	10.32	<0.001	0.9998	18.92	<0.001	0.9997	22.85	<0.001	0.9997	29.31	<0.001	0.9997
89	new	0.9998	10.19	<0.001	0.9998	19.52	<0.001	0.9997	23.11	<0.001	0.9998	29.62	<0.001	0.9997
90	see	0.9998	10.24	<0.001	0.9998	19.25	<0.001	0.9997	23.38	<0.001	0.9997	28.39	<0.001	0.9997
91	good	0.9998	10.28	<0.001	0.9998	19.14	<0.001	0.9998	23.55	<0.001	0.9997	29.07	<0.001	0.9997
92	then	0.9998	10.32	<0.001	0.9998	19.47	<0.001	0.9998	24	<0.001	0.9996	28.33	<0.001	0.9997
93	got	0.9999	10.46	<0.001	0.9998	18.81	<0.001	0.9997	22.76	<0.001	0.9997	28.94	<0.001	0.9997
94	could	0.9998	10.1	<0.001	0.9998	19.03	<0.001	0.9997	23.42	<0.001	0.9997	28.77	<0.001	0.9997
95	other	0.9999	10.46	<0.001	0.9998	19.58	<0.001	0.9998	23.46	<0.001	0.9997	29.48	<0.001	0.9997
96	way	0.9999	10.46	<0.001	0.9998	19.14	<0.001	0.9997	23.11	<0.001	0.9997	29.38	<0.001	0.9996
97	which	0.9998	10.41	<0.001	0.9998	19.25	<0.001	0.9998	23.6	<0.001	0.9996	28.35	<0.001	0.9997
98	much	0.9999	10.55	<0.001	0.9998	19.36	<0.001	0.9998	23.69	<0.001	0.9997	29.24	<0.001	0.9997
99	lot	0.9998	10.32	<0.001	0.9998	19.08	<0.001	0.9997	23.29	<0.001	0.9997	29.16	<0.001	0.9997
100	yes	0.9998	10.32	<0.001	0.9998	19.63	<0.001	0.9998	23.86	<0.001	0.9997	28.93	<0.001	0.9997

t.stat-Length 07	p.value-Length 07	mean-Length 08	t.stat-Length 08	p.value-Length 08	mean-Length 09	t.stat-Length 09	p.value-Length 09	mean-Length 10	t.stat-Length 10	p.value-Length 10	mean-Length 11	t.stat-Length 11	p.value-Length 11	mean-Length 12
NA	NA	0.9955	NA	NA	0.9948	NA	NA	0.9945	NA	NA	0.9939	NA	NA	0.9937
4.11	<0.001	0.9962	5.81	<0.001	0.996	6.75	<0.001	0.9956	9.05	<0.001	0.9951	7.86	<0.001	0.9949
8.77	<0.001	0.9966	8.32	<0.001	0.9963	9.18	<0.001	0.9958	10.51	<0.001	0.9957	12.61	<0.001	0.9954
8.53	<0.001	0.9969	10.97	<0.001	0.9967	12.17	<0.001	0.9964	15.19	<0.001	0.996	14.39	<0.001	0.9957
11.81	<0.001	0.997	11.77	<0.001	0.9968	13.55	<0.001	0.9966	16.63	<0.001	0.9963	16.74	<0.001	0.996
13.19	<0.001	0.9974	14.65	<0.001	0.9971	15.7	<0.001	0.9968	18.06	<0.001	0.9965	19.16	<0.001	0.9964
13.93	<0.001	0.9977	17.59	<0.001	0.9974	18.51	<0.001	0.9973	22.91	<0.001	0.997	22.9	<0.001	0.9966
17	<0.001	0.9978	18.21	<0.001	0.9976	20.7	<0.001	0.9975	24.89	<0.001	0.9973	25.58	<0.001	0.997
17.71	<0.001	0.9981	21.58	<0.001	0.9979	23.01	<0.001	0.9977	26.4	<0.001	0.9974	26.56	<0.001	0.9973
20.64	<0.001	0.9983	22.61	<0.001	0.9981	25.09	<0.001	0.9979	27.77	<0.001	0.9977	29.26	<0.001	0.9976
20.83	<0.001	0.9984	23.95	<0.001	0.9981	25.15	<0.001	0.998	29.32	<0.001	0.9979	31.22	<0.001	0.9978
22.79	<0.001	0.9985	24.59	<0.001	0.9984	27.9	<0.001	0.9982	31.18	<0.001	0.9981	33.59	<0.001	0.9979
22.54	<0.001	0.9985	24.73	<0.001	0.9984	28.02	<0.001	0.9983	31.89	<0.001	0.9982	34.38	<0.001	0.998
23.3	<0.001	0.9986	25.51	<0.001	0.9983	27.69	<0.001	0.9984	33.03	<0.001	0.9981	33.52	<0.001	0.9981
23.23	<0.001	0.9986	26.34	<0.001	0.9984	28.17	<0.001	0.9984	32.92	<0.001	0.9983	36.02	<0.001	0.9982
23.16	<0.001	0.9987	27.67	<0.001	0.9985	29.71	<0.001	0.9984	33.32	<0.001	0.9983	35.8	<0.001	0.9982
24.21	<0.001	0.9987	27.25	<0.001	0.9986	30.25	<0.001	0.9985	34.29	<0.001	0.9984	36.38	<0.001	0.9983
24.99	<0.001	0.9988	27.55	<0.001	0.9987	31.3	<0.001	0.9985	34.54	<0.001	0.9985	37.58	<0.001	0.9984
25.37	<0.001	0.9988	28.25	<0.001	0.9987	31.46	<0.001	0.9986	34.99	<0.001	0.9985	38.15	<0.001	0.9984
25.47	<0.001	0.9989	28.8	<0.001	0.9987	31.37	<0.001	0.9986	35.46	<0.001	0.9985	37.66	<0.001	0.9984
26.23	<0.001	0.9989	29.09	<0.001	0.9988	32.06	<0.001	0.9986	35.18	<0.001	0.9986	39.09	<0.001	0.9984
25.94	<0.001	0.9989	28.8	<0.001	0.9987	31.42	<0.001	0.9987	36.81	<0.001	0.9987	39.85	<0.001	0.9986
26.4	<0.001	0.9989	29.43	<0.001	0.9987	31.92	<0.001	0.9987	36.15	<0.001	0.9987	40.19	<0.001	0.9985
26.05	<0.001	0.999	29.86	<0.001	0.9989	33.39	<0.001	0.9988	37.6	<0.001	0.9987	39.85	<0.001	0.9986
26.36	<0.001	0.9989	29.56	<0.001	0.9989	33.08	<0.001	0.9988	37.64	<0.001	0.9987	40.35	<0.001	0.9986
27.2	<0.001	0.999	30.13	<0.001	0.9989	33.55	<0.001	0.9989	38.1	<0.001	0.9988	40.83	<0.001	0.9986
27.77	<0.001	0.999	29.69	<0.001	0.999	34.56	<0.001	0.9989	38.13	<0.001	0.9987	40.04	<0.001	0.9987
27.06	<0.001	0.999	30.56	<0.001	0.999	34.61	<0.001	0.9989	38.56	<0.001	0.9988	40.96	<0.001	0.9987
27.99	<0.001	0.9991	30.8	<0.001	0.999	34.87	<0.001	0.9989	38.5	<0.001	0.9988	40.54	<0.001	0.9988
27.93	<0.001	0.9991	31.16	<0.001	0.999	34.87	<0.001	0.9989	39.02	<0.001	0.9989	41.75	<0.001	0.9987
27.74	<0.001	0.9992	32.47	<0.001	0.9991	35.67	<0.001	0.9989	38.89	<0.001	0.9989	41.9	<0.001	0.9988
27.82	<0.001	0.9991	31.36	<0.001	0.999	35.18	<0.001	0.999	39.33	<0.001	0.999	42.71	<0.001	0.9989
27.96	<0.001	0.9992	32.49	<0.001	0.9991	36.13	<0.001	0.999	39.98	<0.001	0.9989	42.03	<0.001	0.9989
27.84	<0.001	0.9992	32.35	<0.001	0.9991	36.32	<0.001	0.999	39.57	<0.001	0.999	43.3	<0.001	0.9988
28.62	<0.001	0.9992	32.15	<0.001	0.9991	36.23	<0.001	0.999	39.91	<0.001	0.9989	42.8	<0.001	0.9989
29.68	<0.001	0.9993	33.15	<0.001	0.9991	35.3	<0.001	0.9991	40.32	<0.001	0.999	43.12	<0.001	0.9989
29.49	<0.001	0.9992	32.56	<0.001	0.9991	35.75	<0.001	0.9991	40	<0.001	0.999	43.62	<0.001	0.999
28.62	<0.001	0.9992	32.88	<0.001	0.9992	36.49	<0.001	0.9991	40.23	<0.001	0.999	43.52	<0.001	0.9989
29.68	<0.001	0.9992	32.64	<0.001	0.9991	35.78	<0.001	0.9991	40.31	<0.001	0.999	43.35	<0.001	0.999
29.35	<0.001	0.9992	32.67	<0.001	0.9991	36.43	<0.001	0.9992	41.15	<0.001	0.9991	44.5	<0.001	0.999
28.8	<0.001	0.9993	33.22	<0.001	0.9991	36.36	<0.001	0.9991	40.64	<0.001	0.9991	44.34	<0.001	0.999
29.36	<0.001	0.9992	32.88	<0.001	0.9992	37.05	<0.001	0.9991	41.02	<0.001	0.9991	44.58	<0.001	0.999
29.52	<0.001	0.9993	33.58	<0.001	0.9992	37.32	<0.001	0.9991	40.95	<0.001	0.999	43.61	<0.001	0.9989
29.91	<0.001	0.9992	32.78	<0.001	0.9992	36.98	<0.001	0.9991	41.12	<0.001	0.9991	44.23	<0.001	0.999
28.72	<0.001	0.9993	33.46	<0.001	0.9993	37.48	<0.001	0.9991	40.7	<0.001	0.9991	44.81	<0.001	0.999
29.85	<0.001	0.9992	32.75	<0.001	0.9992	37.5	<0.001	0.9992	41.6	<0.001	0.9991	44.3	<0.001	0.9991
29.94	<0.001	0.9993	33.86	<0.001	0.9992	37.25	<0.001	0.9992	41.14	<0.001	0.9992	45.27	<0.001	0.999
30.16	<0.001	0.9993	33.09	<0.001	0.9992	37.29	<0.001	0.9993	42.83	<0.001	0.9992	45.22	<0.001	0.9991
30.22	<0.001	0.9993	34	<0.001	0.9993	38.15	<0.001	0.9991	40.96	<0.001	0.9991	44.74	<0.001	0.9991
30.33	<0.001	0.9993	33.29	<0.001	0.9993	38.4	<0.001	0.9992	41.28	<0.001	0.9992	45.48	<0.001	0.9991
30.53	<0.001	0.9993	33.49	<0.001	0.9993	38.39	<0.001	0.9992	41.94	<0.001	0.9991	44.93	<0.001	0.9991
30.52	<0.001	0.9993	33.8	<0.001	0.9993	38.29	<0.001	0.9992	41.96	<0.001	0.9991	44.81	<0.001	0.9991
29.99	<0.001	0.9993	33.89	<0.001	0.9993	38.4	<0.001	0.9992	41.61	<0.001	0.9992	45.17	<0.001	0.9991
30.81	<0.001	0.9994	34.33	<0.001	0.9992	37.51	<0.001	0.9993	42.5	<0.001	0.9992	45.32	<0.001	0.9991

30.35	<0.001	0.9994	34.34	<0.001	0.9993	38.47	<0.001	0.9992	42.31	<0.001	0.9992	45.66	<0.001	0.9992
30.62	<0.001	0.9994	34.81	<0.001	0.9992	37.44	<0.001	0.9993	43.28	<0.001	0.9992	45.12	<0.001	0.9992
31.58	<0.001	0.9994	34.37	<0.001	0.9994	38.86	<0.001	0.9992	41.91	<0.001	0.9992	45.36	<0.001	0.9992
31.44	<0.001	0.9994	34.17	<0.001	0.9994	39.02	<0.001	0.9993	42.64	<0.001	0.9993	46.11	<0.001	0.9992
31.56	<0.001	0.9994	34.93	<0.001	0.9994	38.96	<0.001	0.9993	42.4	<0.001	0.9992	45.35	<0.001	0.9992
31.19	<0.001	0.9994	34.43	<0.001	0.9993	38.46	<0.001	0.9993	42.59	<0.001	0.9993	46.57	<0.001	0.9992
30.93	<0.001	0.9994	34.85	<0.001	0.9994	39.45	<0.001	0.9993	42.82	<0.001	0.9992	45.7	<0.001	0.9992
30.6	<0.001	0.9995	35.24	<0.001	0.9994	38.86	<0.001	0.9993	43.06	<0.001	0.9992	45.47	<0.001	0.9992
30.91	<0.001	0.9995	35.29	<0.001	0.9993	38.65	<0.001	0.9993	42.9	<0.001	0.9993	46.41	<0.001	0.9992
31.58	<0.001	0.9995	35.28	<0.001	0.9994	39.29	<0.001	0.9994	43.59	<0.001	0.9993	46.78	<0.001	0.9993
30.67	<0.001	0.9995	35.93	<0.001	0.9994	39.38	<0.001	0.9994	44.24	<0.001	0.9993	46.79	<0.001	0.9993
32.1	<0.001	0.9995	35.46	<0.001	0.9994	39.15	<0.001	0.9994	43.67	<0.001	0.9993	46.48	<0.001	0.9993
31.89	<0.001	0.9994	34.99	<0.001	0.9994	39.69	<0.001	0.9994	43.66	<0.001	0.9993	47.02	<0.001	0.9993
31.11	<0.001	0.9995	35.71	<0.001	0.9994	39.27	<0.001	0.9994	44.04	<0.001	0.9994	47.37	<0.001	0.9993
31.28	<0.001	0.9995	35.08	<0.001	0.9995	40.41	<0.001	0.9994	43.81	<0.001	0.9994	47.35	<0.001	0.9993
31.66	<0.001	0.9995	35.65	<0.001	0.9994	39.89	<0.001	0.9994	44.3	<0.001	0.9993	46.87	<0.001	0.9993
31.38	<0.001	0.9995	36.15	<0.001	0.9995	40.38	<0.001	0.9994	43.98	<0.001	0.9993	47.05	<0.001	0.9994
31.93	<0.001	0.9996	36.61	<0.001	0.9994	39.83	<0.001	0.9994	44.08	<0.001	0.9994	47.37	<0.001	0.9994
32.31	<0.001	0.9996	36.27	<0.001	0.9995	40.26	<0.001	0.9994	44.26	<0.001	0.9993	46.83	<0.001	0.9994
32.27	<0.001	0.9995	36.19	<0.001	0.9995	40.65	<0.001	0.9995	44.65	<0.001	0.9994	48.06	<0.001	0.9993
32.1	<0.001	0.9996	36.8	<0.001	0.9995	40.95	<0.001	0.9994	44.23	<0.001	0.9994	47.67	<0.001	0.9993
31.89	<0.001	0.9996	36.26	<0.001	0.9995	40.4	<0.001	0.9994	44.54	<0.001	0.9994	47.74	<0.001	0.9994
32.13	<0.001	0.9996	36.96	<0.001	0.9996	41.27	<0.001	0.9994	44.18	<0.001	0.9994	47.74	<0.001	0.9994
32.4	<0.001	0.9995	35.83	<0.001	0.9995	40.92	<0.001	0.9994	44.53	<0.001	0.9994	47.73	<0.001	0.9994
32.6	<0.001	0.9996	36.72	<0.001	0.9995	40.55	<0.001	0.9995	44.83	<0.001	0.9995	48.62	<0.001	0.9994
32.17	<0.001	0.9996	36.7	<0.001	0.9996	41.65	<0.001	0.9995	44.64	<0.001	0.9994	48.13	<0.001	0.9994
32.62	<0.001	0.9996	36.68	<0.001	0.9995	40.85	<0.001	0.9995	45.19	<0.001	0.9994	48.1	<0.001	0.9994
33.05	<0.001	0.9996	36.71	<0.001	0.9995	40.51	<0.001	0.9995	44.76	<0.001	0.9995	48.38	<0.001	0.9994
32.85	<0.001	0.9995	36.29	<0.001	0.9996	41.35	<0.001	0.9995	45.31	<0.001	0.9995	48.6	<0.001	0.9995
33.06	<0.001	0.9995	36.27	<0.001	0.9996	41.21	<0.001	0.9995	44.41	<0.001	0.9995	49.06	<0.001	0.9994
32.65	<0.001	0.9996	36.87	<0.001	0.9995	41.15	<0.001	0.9995	44.8	<0.001	0.9995	48.65	<0.001	0.9995
32.23	<0.001	0.9996	36.99	<0.001	0.9996	41.4	<0.001	0.9995	44.82	<0.001	0.9995	48.55	<0.001	0.9995
33.05	<0.001	0.9996	36.89	<0.001	0.9996	41.52	<0.001	0.9995	45.43	<0.001	0.9994	48.26	<0.001	0.9994
33.6	<0.001	0.9996	36.71	<0.001	0.9996	41.4	<0.001	0.9995	44.93	<0.001	0.9995	48.75	<0.001	0.9994
33.36	<0.001	0.9996	36.85	<0.001	0.9996	41.4	<0.001	0.9995	45.44	<0.001	0.9995	48.94	<0.001	0.9994
33.57	<0.001	0.9996	37.04	<0.001	0.9996	41.21	<0.001	0.9995	45.25	<0.001	0.9995	48.69	<0.001	0.9995
33.21	<0.001	0.9996	36.76	<0.001	0.9996	41.63	<0.001	0.9996	45.86	<0.001	0.9995	49.12	<0.001	0.9995
33.65	<0.001	0.9996	36.9	<0.001	0.9996	41.23	<0.001	0.9995	45.52	<0.001	0.9995	48.83	<0.001	0.9995
33.17	<0.001	0.9996	36.82	<0.001	0.9996	41.79	<0.001	0.9996	45.74	<0.001	0.9995	49.18	<0.001	0.9995
33.59	<0.001	0.9996	37.17	<0.001	0.9996	41.44	<0.001	0.9996	45.72	<0.001	0.9995	48.88	<0.001	0.9995
33.67	<0.001	0.9996	37.02	<0.001	0.9996	42.06	<0.001	0.9996	45.92	<0.001	0.9995	49.05	<0.001	0.9995
32.92	<0.001	0.9996	37.09	<0.001	0.9996	41.6	<0.001	0.9995	45.62	<0.001	0.9995	49.24	<0.001	0.9995
33.52	<0.001	0.9996	36.9	<0.001	0.9996	41.92	<0.001	0.9995	45.59	<0.001	0.9995	49.35	<0.001	0.9995
33.63	<0.001	0.9997	37.59	<0.001	0.9996	41.58	<0.001	0.9996	46.29	<0.001	0.9995	49.1	<0.001	0.9996
33.31	<0.001	0.9997	37.85	<0.001	0.9997	42.5	<0.001	0.9996	46.17	<0.001	0.9996	49.73	<0.001	0.9995
33.68	<0.001	0.9996	37.39	<0.001	0.9996	41.79	<0.001	0.9995	45.6	<0.001	0.9995	49.16	<0.001	0.9995

t.stat-Length 12	p.value-Length 12	mean-Length 13	t.stat-Length 13	p.value-Length 13	mean-Length 14	t.stat-Length 14	p.value-Length 14	mean-Length 15	t.stat-Length 15	p.value-Length 15
NA	NA	0.9932	NA	NA	0.9928	NA	NA	0.9922	NA	NA
9.28	<0.001	0.9945	10.43	<0.001	0.9943	11.6	<0.001	0.9939	11.39	<0.001
13.19	<0.001	0.9948	12.69	<0.001	0.9947	14.29	<0.001	0.9946	16.4	<0.001
15.82	<0.001	0.9955	18.85	<0.001	0.9952	18.5	<0.001	0.9949	19.39	<0.001
18.7	<0.001	0.9957	20.25	<0.001	0.9957	22.74	<0.001	0.9951	21.58	<0.001
22.27	<0.001	0.9961	23.93	<0.001	0.9959	25.34	<0.001	0.9957	26.37	<0.001
24.55	<0.001	0.9965	27.02	<0.001	0.9962	27.9	<0.001	0.9961	30.69	<0.001
28.31	<0.001	0.9968	30.28	<0.001	0.9966	31.78	<0.001	0.9964	32.86	<0.001
31.14	<0.001	0.9971	32.61	<0.001	0.9968	33.75	<0.001	0.9968	36.79	<0.001
34.15	<0.001	0.9974	35.57	<0.001	0.9972	38.32	<0.001	0.9972	40.75	<0.001
35.76	<0.001	0.9976	37.55	<0.001	0.9974	39.67	<0.001	0.9974	42.94	<0.001
37.19	<0.001	0.9979	40.56	<0.001	0.9976	42.93	<0.001	0.9975	44.99	<0.001
38.85	<0.001	0.9979	40.61	<0.001	0.9978	44.24	<0.001	0.9976	45.33	<0.001
39.16	<0.001	0.998	41.85	<0.001	0.9978	44.26	<0.001	0.9978	47.42	<0.001
40.01	<0.001	0.998	41.99	<0.001	0.9978	44.62	<0.001	0.9978	47.57	<0.001
39.91	<0.001	0.9981	42.73	<0.001	0.9979	45.73	<0.001	0.9979	49.03	<0.001
41.06	<0.001	0.9981	43.21	<0.001	0.998	46.55	<0.001	0.9979	49.14	<0.001
42.25	<0.001	0.9982	44.37	<0.001	0.9981	48.05	<0.001	0.998	49.48	<0.001
42.28	<0.001	0.9983	44.94	<0.001	0.9981	47.78	<0.001	0.998	49.67	<0.001
42.67	<0.001	0.9983	45.04	<0.001	0.9981	47.15	<0.001	0.998	49.89	<0.001
42.68	<0.001	0.9984	46.02	<0.001	0.9983	49.57	<0.001	0.9982	51.61	<0.001
44.56	<0.001	0.9984	46.06	<0.001	0.9983	50.27	<0.001	0.9981	51.44	<0.001
43.91	<0.001	0.9984	46.67	<0.001	0.9984	51.6	<0.001	0.9982	51.73	<0.001
44.71	<0.001	0.9984	46.79	<0.001	0.9984	50.7	<0.001	0.9983	52.79	<0.001
44.77	<0.001	0.9985	47.71	<0.001	0.9985	51.9	<0.001	0.9983	52.86	<0.001
45.42	<0.001	0.9985	47.76	<0.001	0.9986	53.17	<0.001	0.9984	53.66	<0.001
45.68	<0.001	0.9986	48.56	<0.001	0.9985	52.82	<0.001	0.9984	53.68	<0.001
45.88	<0.001	0.9986	48.96	<0.001	0.9985	53.01	<0.001	0.9985	54.79	<0.001
46.9	<0.001	0.9986	48.4	<0.001	0.9985	52.82	<0.001	0.9985	55.39	<0.001
46.37	<0.001	0.9986	49.17	<0.001	0.9986	52.91	<0.001	0.9985	55.97	<0.001
47.08	<0.001	0.9987	49.59	<0.001	0.9986	53.66	<0.001	0.9985	55.45	<0.001
47.85	<0.001	0.9987	50.07	<0.001	0.9987	54.03	<0.001	0.9986	56.11	<0.001
48.33	<0.001	0.9988	50.53	<0.001	0.9988	55.48	<0.001	0.9987	57.32	<0.001
47.57	<0.001	0.9988	50.34	<0.001	0.9987	54.26	<0.001	0.9986	56.66	<0.001
48.91	<0.001	0.9988	51.52	<0.001	0.9987	54.9	<0.001	0.9987	57.48	<0.001
48.18	<0.001	0.9988	51.45	<0.001	0.9988	55.78	<0.001	0.9987	56.93	<0.001
49.16	<0.001	0.9988	51.28	<0.001	0.9988	56.24	<0.001	0.9987	57.69	<0.001
48.97	<0.001	0.9988	51.47	<0.001	0.9988	55.83	<0.001	0.9988	58.31	<0.001
49.41	<0.001	0.9989	51.98	<0.001	0.9989	56.64	<0.001	0.9988	58.18	<0.001
49.4	<0.001	0.9989	52.05	<0.001	0.9988	56.53	<0.001	0.9988	58.44	<0.001
49.78	<0.001	0.999	53.06	<0.001	0.9989	56.62	<0.001	0.9988	59.02	<0.001
49.3	<0.001	0.999	53.08	<0.001	0.9989	57.15	<0.001	0.9988	59.16	<0.001
48.75	<0.001	0.9989	52.65	<0.001	0.9989	56.84	<0.001	0.9989	59.57	<0.001
49.8	<0.001	0.999	52.83	<0.001	0.9989	56.79	<0.001	0.9989	59.58	<0.001
50.03	<0.001	0.999	53.07	<0.001	0.9989	57.28	<0.001	0.9989	59.56	<0.001
50.43	<0.001	0.999	53.59	<0.001	0.9989	57.75	<0.001	0.9988	58.9	<0.001
49.72	<0.001	0.9989	52.55	<0.001	0.999	58.12	<0.001	0.9988	58.2	<0.001
50.59	<0.001	0.999	53.43	<0.001	0.999	58.17	<0.001	0.9989	59.59	<0.001
51.47	<0.001	0.999	53.41	<0.001	0.999	58.08	<0.001	0.9989	60.32	<0.001
50.52	<0.001	0.999	53.82	<0.001	0.9989	57.57	<0.001	0.9989	59.66	<0.001
51.04	<0.001	0.999	53.47	<0.001	0.999	58.4	<0.001	0.9989	60.2	<0.001
50.87	<0.001	0.999	53.81	<0.001	0.999	58.02	<0.001	0.9989	60.14	<0.001
51.42	<0.001	0.9991	53.97	<0.001	0.9991	59.07	<0.001	0.9989	60.5	<0.001
51.38	<0.001	0.9991	54.35	<0.001	0.9991	59.28	<0.001	0.999	60.91	<0.001

51.85	<0.001	0.9991	54.79	<0.001	0.999	58.83	<0.001	0.999	61.03	<0.001
51.75	<0.001	0.9991	54.33	<0.001	0.9991	59.03	<0.001	0.999	60.87	<0.001
51.77	<0.001	0.9991	54.59	<0.001	0.9991	59.33	<0.001	0.999	61.17	<0.001
52	<0.001	0.9991	54.96	<0.001	0.9991	58.88	<0.001	0.9991	61.75	<0.001
52.58	<0.001	0.9992	55.41	<0.001	0.9991	59.62	<0.001	0.9991	62.12	<0.001
52.16	<0.001	0.9992	55.1	<0.001	0.9991	59.65	<0.001	0.9991	62.2	<0.001
52.35	<0.001	0.9992	55.34	<0.001	0.9991	59.92	<0.001	0.9991	62.52	<0.001
52.36	<0.001	0.9992	55.1	<0.001	0.9992	60.29	<0.001	0.999	61.48	<0.001
52.07	<0.001	0.9992	55.75	<0.001	0.9992	60.62	<0.001	0.9991	62.44	<0.001
52.9	<0.001	0.9992	54.99	<0.001	0.9992	60.6	<0.001	0.9992	62.89	<0.001
53.25	<0.001	0.9992	56.11	<0.001	0.9991	60.13	<0.001	0.9992	62.99	<0.001
52.68	<0.001	0.9992	55.43	<0.001	0.9992	60.69	<0.001	0.9992	63.08	<0.001
52.91	<0.001	0.9992	56.09	<0.001	0.9992	61.03	<0.001	0.9992	62.88	<0.001
52.97	<0.001	0.9993	56.25	<0.001	0.9992	61.08	<0.001	0.9991	62.54	<0.001
53.78	<0.001	0.9992	55.94	<0.001	0.9992	61.22	<0.001	0.9992	62.91	<0.001
53.67	<0.001	0.9992	55.84	<0.001	0.9992	61	<0.001	0.9992	63.13	<0.001
54.08	<0.001	0.9992	56	<0.001	0.9992	61.18	<0.001	0.9992	63.1	<0.001
54.36	<0.001	0.9994	57.59	<0.001	0.9993	61.91	<0.001	0.9992	63.36	<0.001
54.2	<0.001	0.9993	56.92	<0.001	0.9992	61.12	<0.001	0.9992	63.66	<0.001
53.54	<0.001	0.9993	57.05	<0.001	0.9993	61.45	<0.001	0.9992	63.39	<0.001
53.46	<0.001	0.9993	56.83	<0.001	0.9993	61.8	<0.001	0.9993	64.05	<0.001
54.32	<0.001	0.9993	56.95	<0.001	0.9993	61.57	<0.001	0.9992	63.98	<0.001
54.24	<0.001	0.9993	56.9	<0.001	0.9993	61.88	<0.001	0.9992	63.8	<0.001
54.29	<0.001	0.9994	57.6	<0.001	0.9993	61.73	<0.001	0.9993	64.22	<0.001
54.28	<0.001	0.9994	57.61	<0.001	0.9993	62.52	<0.001	0.9993	64.36	<0.001
54.38	<0.001	0.9994	57.67	<0.001	0.9993	62.23	<0.001	0.9993	64.32	<0.001
54.74	<0.001	0.9993	57.32	<0.001	0.9993	61.8	<0.001	0.9993	64.48	<0.001
55.09	<0.001	0.9994	57.89	<0.001	0.9993	62.19	<0.001	0.9993	64.77	<0.001
55.26	<0.001	0.9994	57.59	<0.001	0.9993	61.99	<0.001	0.9993	64.87	<0.001
54.76	<0.001	0.9994	57.63	<0.001	0.9993	62.63	<0.001	0.9993	65.2	<0.001
55.32	<0.001	0.9994	57.76	<0.001	0.9994	62.65	<0.001	0.9994	65.36	<0.001
55.47	<0.001	0.9994	57.96	<0.001	0.9994	63.18	<0.001	0.9994	65.42	<0.001
54.64	<0.001	0.9994	58.04	<0.001	0.9994	62.78	<0.001	0.9994	65.42	<0.001
54.79	<0.001	0.9994	58.5	<0.001	0.9994	63.23	<0.001	0.9993	65.1	<0.001
54.97	<0.001	0.9994	58.2	<0.001	0.9994	62.67	<0.001	0.9994	65.21	<0.001
55.65	<0.001	0.9994	58.5	<0.001	0.9994	63.2	<0.001	0.9994	65.7	<0.001
55.13	<0.001	0.9995	58.63	<0.001	0.9994	63	<0.001	0.9994	65.7	<0.001
55.38	<0.001	0.9994	58.1	<0.001	0.9994	63.23	<0.001	0.9994	65.36	<0.001
55.65	<0.001	0.9995	58.88	<0.001	0.9994	63.66	<0.001	0.9994	65.72	<0.001
55.47	<0.001	0.9995	58.58	<0.001	0.9994	63.46	<0.001	0.9994	65.61	<0.001
56.09	<0.001	0.9995	58.86	<0.001	0.9994	63.27	<0.001	0.9994	65.81	<0.001
55.93	<0.001	0.9995	58.64	<0.001	0.9994	63.74	<0.001	0.9994	66.12	<0.001
55.66	<0.001	0.9995	58.68	<0.001	0.9995	63.93	<0.001	0.9994	65.8	<0.001
56.61	<0.001	0.9995	59.01	<0.001	0.9994	63.29	<0.001	0.9994	65.66	<0.001
55.74	<0.001	0.9995	58.84	<0.001	0.9995	64.13	<0.001	0.9994	66.26	<0.001
56.12	<0.001	0.9995	59.38	<0.001	0.9995	63.96	<0.001	0.9994	66.04	<0.001
